# Immunotherapy and Immune Evasion in Prostate Cancer

**DOI:** 10.3390/cancers5020569

**Published:** 2013-05-24

**Authors:** Archana Thakur, Ulka Vaishampayan, Lawrence G. Lum

**Affiliations:** 1 Department of Oncology, Wayne State University, Detroit, MI 48201, USA; 2 Department of Medicine, Wayne State University, Detroit, MI 48201, USA; 3 Department of Immunology and Microbiology, Wayne State University, Detroit, MI 48201, USA

**Keywords:** prostate cancer, immunotherapy, vaccine, castrate resistant prostate cancer, bispecific antibody

## Abstract

Metastatic prostate cancer remains to this day a terminal disease. Prostatectomy and radiotherapy are effective for organ-confined diseases, but treatment for locally advanced and metastatic cancer remains challenging. Although advanced prostate cancers treated with androgen deprivation therapy achieves debulking of disease, responses are transient with subsequent development of castration-resistant and metastatic disease. Since prostate cancer is typically a slowly progressing disease, use of immune-based therapies offers an advantage to target advanced tumors and to induce antitumor immunity. This review will discuss the clinical merits of various vaccines and immunotherapies in castrate resistant prostate cancer and challenges to this evolving field of immune-based therapies.

## 1. Introduction

Despite current advances, metastatic prostate cancer (PC) remains an incurable malignancy. Hormone therapy that suppresses testosterone is effective for a finite period of time, after which almost all patients develop castrate resistant prostate cancer (CRPC) [1]. Prostate cancer is a molecularly heterogeneous disease, which may arise from more than twenty different clonal subtypes [2]. Even though there are prostate associated antigens such as prostate-specific antigen (PSA), prostate-specific membrane antigen (PSMA), prostate stem-cell antigen, and prostatic acid phosphatase (PAP), they are weak or non-immunogenic self-antigens [3,4,5,6,7,8,9]. Prostate cancer, typically a slowly progressing, asymptomatic disease, is an attractive target for immune-based therapies with the large window of opportunity for multiple vaccinations or boosting for the development of antitumor immune responses [10,11,12]. One of the promising approaches for cancer immunotherapy is dendritic cell (DC)-based vaccination to initiate antigen-specific antitumor immune responses with minimal toxic side effects [10]; unfortunately, results from clinical trials showed mixed responses [13]. Given the limited life expectancy and the significant morbidity of metastatic castration-resistant prostate cancer (mCRPC) there continues to be a “need” for novel non-toxic approaches to decrease pain, delay morbidity, improve quality of life, and improve life expectancy in metastatic CRPC. Between 2010 and 2012, four new agents were approved by the US Food and Drug Administration (FDA) for patients with metastatic CRPC. All of these new agents (sipuleucel-T, abiraterone acetate, enzalutamide, and cabazitaxel) have been shown to improve overall survival in patients with metastatic CRPC. However, complete remissions are rare, and further evaluation of novel agents to achieve this elusive goal should continue. The approval of sipuleucel-T for prostate cancer is a milestone for cell-based immunotherapy. Further enhancements in the clinical efficacy of current approaches may be accomplished by combining both T cell- and antibody-based vaccination strategies with the current standard of care regimens. This review will focus on immune based or immune modulating therapies.

## 2. DC-Based Vaccination Strategies

Dendritic cells are professional antigen-presenting cells (APCs) and key regulators of T- and B-cell immunity due to their unique ability to take up, process and present antigens to T cells [14]. In the 1980s it was established that antibodies enhance specific T-cell responses by promoting Fc receptor (FcR)-mediated recognition of opsonized antigens by cross presentation mediated by APCs wherein DC were used as vaccine adjuvants [15]. These findings formed the basis for the targeted delivery of antigens by DC in the context of MHC class I and II surface molecules to enhance T-cell-mediated antitumor immune responses. Exploitation of these antitumor effects of DC resulted in the development of vaccination strategies [16,17,18]. In the prostate cancer setting, approaches such as peptide vaccines, virally packaged antigens, and DNA-based antigen-expressing vectors have been used to pulse the DCs that can promote tumor-specific T-cell responses [18,19,20,21,22,23,24,25]. In addition to loading tumor associated antigens (TAA), other tumor modulating agents such as granulocyte macrophage colony stimulating factor (GM-CSF) and toll like receptor (TLR) agonists (Bacillus Calmette-Guérin [BCG] and CpG) were either used as adjuvants or engineered to enhance antigen presentation by APCs [26,27]. 

### 2.1. Sipuleucel-T

Given that PAP expression is essentially restricted to prostate tissue [28,29], PAP expression on metastatic prostate cancer makes it a very specific target [28]. Provenge^®^ (Sipuleucel-T, Dendreon Corp, Seattle, WA, USA) is an autologous active cellular immunotherapy. The target antigen PA2024 used to prepare Provenge^®^ is a fusion protein consisting of full-length human prostatic acid phosphatase (PAP) and full length human GM-CSF. Sipuleucel-T is prepared by culturing freshly obtained leukapheresis peripheral blood mononuclear cells (PBMC) with PA2024 for 36–44 h at 37 °C. A complete course of sipuleucel-T therapy consists of three freshly prepared doses of sipuleucel-T administered via intravenous (IV) infusion at approximately 2-week intervals. In a placebo-controlled phase III study, the efficacy of sipuleucel-T was evaluated. In this study (protocol D9901), 127 patients with asymptomatic metastatic hormone refractory prostate cancer (HRPC) were randomly assigned in a 2:1 ratio to receive three infusions of sipuleucel-T (n = 82) or placebo (n = 45) every 2 weeks. On disease progression, placebo patients could receive a product made from frozen leukapheresis cells (APC8015F). All patients were followed for survival for 36 months, 115 of 127 patients had progressive disease at the time of data analysis. The median for time to disease progression (TTP) for sipuleucel-T was 11.7 weeks compared with 10.0 weeks for placebo (*p* = 0.052, log-rank; hazard ratio [HR], 1.45; 95% CI, 0.99 to 2.11). Median survival was 25.9 months for sipuleucel-T and 21.4 months for placebo (*p* = 0.01, log-rank; HR, 1.70; 95% CI, 1.13 to 2.56). While the improvement in the primary end point TTP did not achieve statistical significance, this study suggested that sipuleucel-T may be providing a survival advantage to asymptomatic CRPC patients [30]. A second contemporaneous study, D9902A, in which enrollment was 44 discontinued early (N = 98), showed a trend towards improved survival, which did not reach statistical 45 significance.The treatment effect remained strong after performing adjustments for imbalances in baseline prognostic factors, post study treatment chemotherapy use, and non-prostate cancer-related deaths and suggested a favorable risk-benefit ratio for sipuleucel-T in patients with advanced prostate cancer [23]. The most common adverse events associated with treatment were chills, pyrexia, headache, asthenia, dyspnea, vomiting, and tremor. These events were primarily grade 1 and 2 that lasted for 1 to 2 days. The integrated results of D9901 and D9902A demonstrated a survival benefit for patients treated with sipuleucel-T compared with those treated with placebo [23,31]. Another randomized, double-blind, placebo-controlled phase III trial D9902B (the IMPACT [Immunotherapy for Prostate Adenocarcinoma Treatment]) was designed with OS as the primary end point. This trial enrolled 512 men at a ratio of two to one. The study recapitulated the results of D9901, showing a 4.1-month improvement in median OS (25.8 *versus* 21.7 months) with no effect on TTP (14.6 *versus* 14.4 weeks). After the OS benefit was confirmed in a larger phase III placebo controlled trial, Provenge^®^ therapy was approved by the FDA in April 2010 for the treatment of asymptomatic or minimally symptomatic metastatic CRPC. 

Recently, Sheikh *et al.* analyzed the data for immunological responses to sipuleucel-T therapy and correlated the immunological responses with overall survival (OS) by assessing antigen-specific cellular and humoral responses [32]. Peripheral immune responses were measured in a subset of consented subjects enrolled in the IMPACT study (n = 237). Authors show that APC activation occurred in the first dose and increased in the second and third dose preparations. Cumulative APC activation and APC number correlated with OS (*p* < 0.05). Interferon gamma (IFNγ) enzyme-linked immunosorbent spots (ELISPOT) evaluated at 0, 2, and 4 weeks after treatment showed antigen-specific immune responses in 78.8% of monitored subjects and their presence correlated with OS (*p* = 0.003). These data suggest that large majority of patients not only showed induction of immune responses but immune responder patients showed positive correlation with OS. Induction of antigen-specific immune activation may be the mechanism by which sipuleucel-T may prolong OS [32]. 

### 2.2. GM-CSF-Modified Tumor Cell Vaccines

GVAX^®^ (Cell Genesys, Inc., South San Francisco, CA, USA) vaccines are comprised of genetically modified tumor cells engineered to secrete GM-CSF. GVAX was constructed from two allogeneic cell lines, LN-CaP and PC-3. These cell lines were selected since they represent a broad antigenic spectrum of prostate cancer. The PC-3 cell line was derived from a prostate cancer bone metastasis and is hormone-refractory, which is the hallmark of the lethal phenotype of prostate cancer [33,34]. The LnCaP is a hormone sensitive cell line, which was developed from a prostate cancer metastasis to a lymph node, expresses a number of restricted differentiation antigens, including prostate-specific antigen (PSA), prostate-specific membrane antigen and a mutant androgen receptor [34]. These two cell lines were genetically modified to secrete GM-CSF. GM-CSF is a potent cytokine activator of APCs, and plays an important part in breaking tolerance and the development of antitumor immune responses [34].

A single-institution phase I/II trial was done in hormone therapy–naïve patients with prostate-specific antigen (PSA) relapse following radical prostatectomy and absence of radiologic metastases [35]. Treatments were administered weekly via intradermal injections of 1.2 × 10^8^ GM-CSF gene–transduced, irradiated, cancer cells (6 × 10^7^ LNCaP cells and 6 × 10^7^ PC-3 cells) for 8 weeks. The study enrolled 21 patients. Toxicities included local injection-site reactions, pruritus, and flu-like symptoms. Data analysis for immunological responses showed recruitment of CD1a^+^ dendritic cells and CD68^+^ macrophages at injection site in biopsies samples. Patients developed new polyclonal antibodies reactive against antigens present in LNCaP or PC-3 cells post treatment [36]. A partial PSA response in 1 of 21 patients and a reduction in PSA velocity post treatment in 16 of 21 patients provide preliminary evidence of clinical antitumor activity [35]. A second trial was conducted in 55 chemotherapy-naïve patients with hormone-refractory prostate cancer (HRPC). HRPC patients with radiologic metastases (*n* = 34) or rising PSA (*n* = 21) received a prime dose of 500 million cells and 12 boost doses of either 100 million cells (low dose) or 300 million cells (high dose) biweekly for 6 months. End points were changes in PSA, TTP, and overall survival. Median overall survival was 26.2 months (95% confidence interval, 17, 36) in the radiologic group: 34.9 months (8, 57) after treatment with the high dose (*n* = 10) of immunotherapy and 24.0 months (11, 35) with the low dose (*n* = 24). The most common adverse events were injection site reaction and fatigue, no dose-limiting or autoimmune toxicities were seen. These results suggest that this GM-CSF–secreting, allogeneic cellular immunotherapy is well tolerated and may have clinical activity in patients with metastatic HRPC [36]. This vaccine was subsequently modified to increase GM-CSF production. The safety and activity of this modified product was evaluated in a phase I–II, multicenter, open-label study in patients with metastatic CRPC. Eighty men with progressive asymptomatic, chemotherapy-naive PC with castration-resistant disease were treated with different dose levels of the vaccine product [37]. The median survival time was 35 months in the high-dose group, 20 months in the mid-dose, group, and 23.1 months in the low-dose group. PSA stabilization occurred in 15 patients (19%). The most common adverse effect was injection-site erythema and a maximal tolerated dose was not established. The proportion of patients who generated an antibody response to one or both cell lines increased with dose and included 10 of 23 (43%) in the low-dose group, 13 of 18 (72%) in the mid-dose group, and 16 of 18 (89%) in the high-dose group [37]. 

Phase III trial of GVAX in asymptomatic CRPC patients (VITAL 1) was designed to enroll 600 patients with superiority in overall survival as primary endpoint compared to chemotherapy (docetaxel/prednisone) arm [38]. The study completed accrual of 626 patients with more than 45% of patients with Gleason score > 8. The median follow up period was 66 weeks. The study was closed early due to disappointing results of interim analysis. The median survival was 20.7 months on GVAX and 21.7 months on docetaxel plus prednisone arm, with hazard ratio 1.03 during 66 weeks of follow up period. The toxicity was much less in GVAX arm compared to docetaxel plus prednisone arm. In the subset of men with Halabi predicted survival (HPS) >18 months (*n* = 264), median survival was prolonged on GVAX (29.7 months) compared to docetaxel plus prednisone (27.1 months) suggesting that an immunotherapy may take longer to induce favorable effect [38]. The lack of efficacy of GVAX may be due to the study design such as using chemotherapy as a comparator arm and inclusion of patients with more aggressive disease (>45% patients had >8 Gleason score). Vaccines are more likely to be efficacious in patients with less aggressive disease and in those who had prior chemotherapy to reduce the tumor burden. A recent study reported that treatment with GVAX plus ipilimumab is feasible and safe in mCRPC patients. Improvement in bone scan, and tumor regression on CT scan suggest that this combination of immunotherapy has clinical activity in mCRPC and provides rationale for combination therapy [39]. 

### 2.3. ProstVac-VF

ProstVac-VF, a PSA targeted therapeutic vaccine, is a combination of recombinant vaccinia and fowlpox viruses vaccine that delivers PSA along with three costimulatory signals (known as Tricom) [24] to enhance antigen uptake by DC and subsequent antigen presentation to T-cells. Both vectors contain the transgenes for prostate-specific antigen (PSA) and three T-cell co-stimulatory molecules (B7.1, ICAM-1, and LFA-3; termed Tricom). Use of two viruses in ProstVac-VF vaccine was to avoid the effect of neutralizing antibodies formed after immunization with the first vaccinia virus; in this heterologous prime/boost strategy the subsequent boost is given with the fowlpox virus. The interaction of these APCs with T cells initiated a targeted immune response and T cell-mediated tumor cell destruction. Phase I trial evaluated the clinical safety of this vaccine approach using recombinant vaccinia virus (prime) and recombinant fowlpox virus (boost) in combination with GM-CSF in 15 metastatic prostate cancer patients. Initial clinical studies showed that vectors were safe, induction of PSA-specific immune responses, and reduction in PSA levels [40,41,42,43]. Based on the safety and preliminary immunogenicity results of this trial, a randomized phase II study of prostate specific antigen/tricom vaccines was recommended in patients with less advanced prostate cancer. A phase II randomized clinical trial with ProstVac-VF was conducted by the Eastern Cooperative Oncology group. Patients were randomly assigned vaccine combination, Arm (A) received four rF-PSA vaccines, Arm (B) received three rF-PSA vaccines followed by a single rV-PSA vaccine and, Arm (C) received a single rV-PSA vaccine followed by three rF-PSA vaccinations. The major end point was PSA response at 6 months, and PSA-specific T-cell responses. The prime/boost schedule was well tolerated with negligible toxicity. Overall, of all the eligible patients, 45.3% of men remained free of PSA progression at 19.1 months, 78.1% of the men demonstrated clinical progression-free survival (PFS) and 46% of men demonstrated an increase in PSA-reactive T-cells [44]. 

ProstVac-VF treatment was also evaluated for prolongation of PFS and OS in a randomized, controlled, and blinded phase II study in 125 patients who had minimally symptomatic mCRPC. Patients were allocated (2:1) to ProstVac-VF plus GM-CSF or to control empty vectors plus saline injections. Eighty-two patients received ProstVac-VF and 40 received control vectors. The primary end-point was progression free survival (PFS), which was similar in the two groups (*p* = 0.6). However, at 3 years post study, ProstVac-VF patients had a better OS with 25 (30%) of 82 alive *versus* 7 (17%) of 40 controls, prolonged median OS by 8.5 months (25.1 *versus* 16.6 median OS months for controls) in men with mCRPC [45]. Based on an 8.5-month improvement in median overall survival observed in this trial, a randomized double-blind phase III trial has been designed which will compare the effect of ProstVac-VF with or without GM-CSF *versus* placebo on overall survival in men with minimally symptomatic mCRPC and will enroll 1,200 patients (ClinicalTrials.gov Identifier: NCT01322490). In non-mCRPC there have been three ongoing phase II trials that are evaluating ProstVac-VF alone or in combination with chemo- and radioimmunotherapy (ClinicalTrials.gov Identifiers: NCT00450463, NCT01145508, and NCT00450619). 

A Phase I clinical trial of combination therapy of ipilimumab and a PSA-Tricom in metastatic castration-resistant prostate cancer showed evidence of clinical benefit with the median overall survival of longer than 34 months and development of specific immune responses in six out of 30 patients. Study provides the rationale to combine two forms of modern immune-based therapies without more clinically significant or synergistic toxic effects [46,47].

## 3. DNA Vaccines

Immunizations with plasmid DNA encoding tumor-associated antigens has been shown to induce potent humoral and cellular immune responses [48,49,50,51]. A preclinical study showed that injection of a DNA vaccine encoding full-length prostatic acid phosphatase (PAP) antigen elicited an antigen-specific CD8^+^ T cells in rodents [18], led to a phase I/IIa trial with a DNA vaccine encoding human PAP in patients with stage D0 prostate cancer with the goal to elicit a sustainable immune response, able to eradicate a tumor or at least, restrain its growth [52]. In this trial, 22 patients were treated in a dose-escalation manner with 100, 500, or 1,500 μg of plasmid DNA, co-administered intradermally with 200 μg GM-CSF, six times at 14-day intervals. Three of 22 (14%) patients developed PAP-specific IFNγ-secreting CD8^+^ T-cells immediately after the treatment course. Nine of 22 (41%) patients developed PAP-specific CD4^+^ and/or CD8^+^ T-cell proliferation. Antibody responses to PAP were not detected. Overall, the PSA doubling time was observed to increase from a median 6.5 months pretreatment to 8.5 months on-treatment (*p* = 0.033), and 9.3 months in the 1-year post-treatment period (*p* = 0.054). This study established that a PAP encoding DNA vaccine is safe and elicits an antigen-specific T-cell response [52]. Immunologic efficacy of PAP encoding DNA vaccine was also reported by Becker *et al.*, this study showed that antigen-specific cytolytic T-cell responses were amplified after immunization in seven of 12 HLA-A2 expressing individuals, and that multiple immunizations seemed necessary to elicit PAP-specific IFNγ Elispots [53]. These data suggest that DNA vaccines targeting PAP could potentially be combined in heterologous immunization strategies with other vaccines to further augment PAP-specific T-cell immunity [53].

## 4. Armed Activated T Cell (ATC) Therapy-HER2 “Positive” CRPC as Targets

In prostate cancer, Her2/*neu* (HER2) over expression is reported to high [54,55,56]. Patients with HER2 positive (2+ or higher on IHC) cancers have better survival and lower relapse rates as compared to the HER2 negative prostate cancers [57]. Over-expression of HER2 in CRPC patients makes it an ideal target for anti-CD3 activated T cells (ATC) armed with anti-CD3 × anti-Her2 bispecific antibody (Her2Bi). Our approach combines the non-MHC-restricted cellular cytotoxicity mediated by anti-CD3 activated T cells (ATC) coated with the bispecific antibodies. One end of bispecific antibody binds to T cells through anti-CD3 and other end to the Her2/*neu* on the tumor cells through anti-Her2 antibodies. After arming with Her2Bi, every T cell is transformed into a specific cytotoxic T cell directed at tumor cells. Our preclinical studies show that ATC armed with Her2Bi exhibited high levels of non-MHC restricted cytotoxicity directed at PC-3, DU-145, and LNCaP prostate cancer cell lines produced tumoricidal cytokines such as interferon γ (IFNγ), tumor necrosis factor α (TNFα), and GM-CSF as well as MIP-1alpha and RANTES [58,59,60]. Our findings suggest that Her2Bi-armed ATC therapy may be an effective, nontoxic, tumor-specific treatment for Her2-positive CRPC.

Metastatic patients with higher serum HER2 levels had a shorter time to recurrence when compared to those with lower levels [57,61]. Our phase I trial in seven patients with CRPC established the safety of Her2Bi-armed ATC infusions [62]. The PSA levels decreased in three of 7 (43%) patients and one of seven had a >50% decline in PSA below baseline levels that persisted more than 4 months. There was a decrease in narcotic use in two of the 7 (28.5%) men possibly due to decreased bone pain. Evaluation of immune responses in our phase I clinical trial CRPC patients suggest that infusions of Her2Bi-armed ATC induce robust long-lasting anti-tumor responses. These data suggest that aATC therapy either alone or in combination with other vaccines may provide additional benefit to metastatic PC patients. 

## 5. Chemotherapies as Immune Modulators

Cancer chemotherapy and radiotherapy cause a direct cytotoxic effect on tumor cells; dying tumor cells release molecules that promote the activation and the functional maturation of the most potent antigen-presenting dendritic cells [63,64,65]. One study showed the immunostimulatory properties of dying tumor cells after chemotherapy (chemoT) or radiation therapy and suggested that inflammation and TLR signaling play important roles in cancer chemotherapy [66]. Crosspresentation of antigens from apoptotic tumor cells in the context of MHC class I required TLR4 and MyD88 to generate antitumor cytotoxic T cell (CTL) responses triggered by the nuclear protein high-mobility group box 1 protein (HMGB1). Patients with breast cancer who carry a TLR4 loss-of-function allele relapse more quickly after radiotherapy and chemotherapy than those carrying the normal TLR4 allele. These results describe a clinically relevant immunoadjuvant pathway triggered by chemotherapy induced tumor cell death [66].

Combinations of chemotherapy and immunotherapy show induction of immune responses in patients who were given lower, more frequent doses of docetaxel (without daily steroids) combined with vaccine [67]. However, studies that combine vaccine with higher doses of docetaxel are challenged by the lymphodepleting properties of chemotherapy [67]. In Eastern Cooperative Oncology Group trial (E1809) immunotherapy (ProstVac-VF) was administed before chemotherapy with the idea to avoid the immunosuppressive effects of chemotherapy and create a proinflammatory microenvironment in which tumor-cell destruction by chemotherapy can be augmented by immune-mediated tumor lysis using comparator arm as chemotherapy alone [68]. However, this trial was closed early due to poor accrual (after enrolling only 10 out of 144 patients) [68]. 

Furthermore, a randomized phase II clinical trial was designed to determine if a poxviral vaccine encoding PSA can induce a PSA-specific T-cell response when combined with radiotherapy in patients with clinically localized prostate cancer [69]. Thirty patients were randomized in a 2:1 ratio into vaccine plus radiotherapy or radiotherapy-only arms. Seventeen of 19 (89%) patients in the combination arm completed all eight vaccinations and 13 of these 17 (76%) patients had increases in PSA-specific T cells of at least 3-fold *versus* no detectable increases in the radiotherapy-only arm (*p* < 0.0005). This vaccine regimen can be safely given in patients undergoing radiation therapy for localized prostate cancer, with the majority of patients generating a PSA-specific cellular immune response to vaccine [69]. 

The VITAL 2 phase III trial compared GVAX + docetaxel to docetaxel plus prednisone alone arm in symptomatic CRPC patients. This trail enrolled 408 of 600 planned patients but was closed early due to an increased number of deaths [70,71]. Survival curve showed inferior survival for GVAX + docetaxel arm compared to docetaxel plus prednisone arm with median survival of 12.2 and 14.1 months and hazard ratio of 1.7, however, longer follow up showed reduction in hazard ratio of 1.4 [70,71]. In this trial there may be several reasons that may have lead to the lack of efficacy of GVAX, (a) an unknown interaction of GM-CSF with docetaxel; (b) GM-CSF in GVAX may stimulate myeloid derived suppressor cells leading to the endogenous immune suppression as a result of docetaxel and GM-CSF interaction; (c) reduced dose of docetaxel and treatment schedule may have caused the increased rate of disease progression. A careful examination of deficiencies and flaws in the study design of this phase III trail may provide insight into designing future studies with better outcome. However, it is important to note that there is often a delayed effect seen in immunotherapy studies and since both GVAX phase III trials were terminated early, it is likely that in the early follow up period outcome appeared worse.

## 6. Antibody Based Immunotherapy Targeting Checkpoint Inhibitors

Cytotoxic T-lymphocyte–associated antigen-4 (CTLA-4) is the best characterized regulatory molecule of the immunoglobulin superfamily [72,73]. CTLA-4 and programmed death-1 (PD-1) are the immunologic regulators which prevent immune-mediated damage to normal tissues [74,75], but on the flipside these innate immune checkpoints can also inhibit immune responses. Both CTLA-4 and PD-1 are upregulated with T-cell activation, and the ligands for PD-1 (PD-L1, PD-L2) are often expressed by tumors. Therefore, to design the successful therapies, immunosuppressive mechanisms have to be targeted simultaneously [76]. CTLA-4 blockade enhances T cell activation and memory against a poorly immunogenic spontaneous murine tumor and generates antitumor T-cell responses in early stages of tumor growth [77]. Similarly, the combination of CTLA-4 blockade and a vaccine consisting of GM-CSF-expressing cancer cells resulted in regression of parental tumors, despite the ineffectiveness of either treatment alone in murine model [78]. Collectively, these preclinical experiments suggest that appropriate manipulation of T cell costimulatory and inhibitory signals may provide a basis for CTLA-4 based prostate cancer immunotherapy. 

The safety and activity of anti-CTLA-4 Ab (ipilimumab; Bristol-Myers Squibb) alone or with a single dose of docetaxel in HRPC was evaluated by Small *et al*. [79]. Chemotherapy naïve patients (*n* = 43) with HRPC were treated; 23 were in arm A (ipilimumab at 3 mg/kg q 4 weeks × 4 doses) and 20 in arm B (ipilimumab as in Arm A and one dose of 75 mg/m^2^ of docetaxel on day 1). Six patients, three in each arm, demonstrated a decrease in PSA of >50%. Three patients, two in arm A, and one in arm B had confirmed PSA responses with durations of 79+, 169+, and 280 days, respectively [79]. Another study investigated the diversity of Ab responses modulated by treatment with CTLA-4 blockade and GM-CSF in a phase I trial where a combination of ipilimumab and GM-CSF was administered to patients with metastatic CRPC who had not received any prior chemotherapy or immunotherapy [80,81]. Authors demonstrated that blocking of immune checkpoint modulates Ag-specific responses to both individualized and shared Ags, some of which can mediate anti-tumor responses. In addition, they showed that clinical responders develop Ag-specific immune responses distinct from clinical non-responders [81]. In addition anti-CTLA-4 monotherapy, trials are ongoing with anti-CTLA-4 (ipilimumab) combined with ProstVac-VF [82] or a GM-CSF-secreting whole tumor cell vaccine (GVAX; BioSante Pharmaceuticals) in prostate cancer [83].

Like CTLA-4, PD-1 is also an inhibitory receptor expressed on activated T cells and known to inhibit antitumor immunity [84]. CD8^+^ T cells that infiltrate prostate and melanoma tumors express high levels of PD-1 and have impaired effector functions, moreover, B7-H1/PD-1 forms a molecular shield to prevent destruction by CTLs [85] suggesting that reversal of PD-1 signaling in those cells can have direct effects on the tumor cell killing [86,87]. Study reported by Hamanishi *et al*. showed a significant inverse correlation between PD-L1 expression and the intraepithelial CD8^+^ T lymphocyte count, suggesting that PD-L1 on tumor cells directly suppresses antitumor CD8^+^ T cells [87]. The diversity of CD8^+^ TCR beta chain variable region (Vbeta) gene sequences in both the peripheral blood and prostates of cancer patients exhibited restricted TCR Vbeta gene usage in CD8^+^ prostate infiltrating lymphocytes and express high levels of the inhibitory receptor PD-1. These data suggest that PD-1 blockade may be useful in immunotherapy for prostate cancer [88].

Antibodies to PD-1 have demonstrated efficacy in a number of malignancies in phase I clinical trials, including prostate cancer. The safety and tolerability of anti-PD-1 blockade in patients with treatment-refractory solid tumors was reported by Brahmer *et al.* Thirty-nine patients with advanced metastatic melanoma, colorectal cancer (CRC), CRPC, non-small-cell lung cancer (NSCLC), or renal cell carcinoma (RCC) received a single intravenous infusion of anti-PD-1 (MDX-1106; Bristol-Myers Squibb) in dose-escalating six-patient cohorts at 0.3, 1, 3, or 10 mg/kg, followed by a 15-patient expansion cohort at 10 mg/kg. Patients with evidence of clinical benefit at 3 months were eligible for repeated therapy [89]. Blocking the PD-1 immune checkpoint with intermittent antibody dosing is well tolerated and associated with evidence of antitumor activity. A phase I trial showed objective responses in a number of tumors with PD-1 antagonists [90], a partial response was seen in one of 15 (6.7%) patients, and stable disease (>4 months) was seen in three of 15 (20%) patients with CRPC [90], immune-related toxicities seem to be a more benign than CTLA-4 blockade [91]. 

In summary, the IT experience in metastatic PC highlights the following: (1) Approval of sipuliucel-T and results of randomized phase II clinical trial with ProstVac-VF suggest that IT may be effective against prostate cancer (2) Much less toxicity and adverse events indicate that IT is better tolerated than the current chemotherapy regimens (3) Study design and identification of patient subsets responding to IT remains a challenge (lessons from Phase III trials with GVAX); (4) Targeting of immunosuppressive tumor microenvironment are needed prior to the design and beginning of large randomized trials (such as combining vaccines with immunotherapies targeting checkpoint inhibitors). 

## 7. The Mechanisms of Immune Evasion/Immunosuppression

Tumors have developed mechanisms to evade the immune system [92]. The tumor microenvironment can also support the recruitment and expansion of myeloid derived suppressor cells (MDSC), T regulatory cells (Tregs), tumor associated macrophages (TAMs) that can inhibit effector T-cell functions [93,94]. In addition, tumor-related factors can impede the maturation of DCs through secretion of immunosuppressive cytokines, and mutated or lack of expression of immunomodulatory molecules [95]. Molecular targets regulating immune suppression include arginase, nitric oxide synthase, indoleamine-2,3-dioxygenase (IDO), and signal transducers and activators of transcription (STAT) [96,97,98]. Both STAT-1 and STAT-3 signaling have been implicated in tumor development. Studies have shown tumor escape mechanisms in STAT-1^−/−^ mice, and STAT-3 signaling was identified in the inhibitory effects of IL-10 on DC maturation and migration, and impairment of CD4^+^ T-cell function [97]. 

### 7.1. Myeloid Derived Suppressor Cells (MDSC)

MDSCs are an important cell subset that contributes to an immunosuppressive tumor microenvironment [99,100]. MDSC accumulation and activation are driven by multiple factors, many of which are identified with chronic inflammation [101,102]. The expansions of MDSCs are associated with several inflammatory mediators, and STAT3 is arguably the main transcription factor that regulates the expansion of MDSCs [103]. Investigation of changes in the levels of circulating MDSC with progression of PC and after the immunotherapy [104] showed high percentage of CD14^+^/HLA-DR^l^°^/−^ monocytic MDSC in treated PC (30.7 ± 15.0% of CD14^+^ cells) compared to untreated PC (10.6 ± 14.3%, *p* = 0.0001) patients. These CD14^+^/HLA-DR^l^°^/−^ monocytes were able to suppress immune cell functions *in vitro*. Elimination of these MDSC may thus significantly improve antitumor responses and enhance effects of cancer immunotherapy [104,105]. The effect of gemcitabine on the number of (Gr-1+/CD11b+) cells was studies in the spleens of animals bearing large tumors derived from five cancer lines grown in both C57Bl/6 and BALB/c mice [106,107]. This study showed that gemcitabine, given at a dose similar to the dose used in patients, was able to dramatically and specifically reduce the number of MDSC in the spleens of animals bearing large tumors with no significant reductions in CD4^+^ T cells, CD8^+^ T cells, NK cells, macrophages, or B cells. The loss of myeloid suppressor cells was accompanied by an increase in the antitumor activity of CD8^+^ T cells and activated NK cells [106,107]. 

In a randomized, double-blind, placebo-controlled phase II trial, Pili *et al.* [108] investigated the activity of the novel antitumor agent tasquinimod (TASQ), which targets the S100A9 receptor expressed on MDSC, in men with metastatic castration-resistant prostate cancer (CRPC) and minimal symptoms. In this study, patients were assigned (at a ratio of two to one) to either oral once-daily TASQ 0.25 mg/d escalating to 1.0 mg/d over 4 weeks or placebo. The primary end point was the proportion of patients without disease progression at 6 months. Two hundred one evaluable patients with balanced baseline characteristics, 134 were assigned to TASQ and 67 to placebo. For TASQ group the 6-month progression-free proportion was 69% (*p* < 0.001)compared to 37% in placebo group, and median progression-free survival (PFS) was 7.6 *versus* 3.3 months (*p* = 0.0042). This study showed that TASQ significantly slowed disease progression and improved PFS in patients with metastatic CRPC with an acceptable AE profile. Since one of the molecular targets for TASQ is a receptor S100A9 expressed on MDSCs [109,110], authors suggest that the antiangiogenic [110] and antimetastatic properties of TASQ are mediated through modulation of MDSC activity within the tumor microenvironment.

We have recently shown a significant decrease in MDSC populations in the presence of ATC armed with either Her2Bi or EGFRBi (anti-CD3 × anti-EGFR bispecific antibodies) in our *in vitro* 3D culture model. These data suggest that aATC can suppress MDSC differentiation and attenuation of their suppressive activity through down regulation of COX2, PGE_2_ and ARG1 [111] that is potentiated in presence of Th_1_ cytokines and chemokines (IFN-γ, IL-2, CXCL9 and CXCL10) [112]. Immunotherapeutic strategies that can target MDSC and tumor cells simultaneously may improve the antitumor efficacy of the treatment. 

### 7.2. T-Regulatory Cells (Tregs)

Tregs (5–10% of the peripheral CD4^+^ T cells) are responsible for peripheral tolerance to self-antigens [78] while absence of Treg favors autoimmunity. These regulatory T cells also play a critical role in suppressing immune responsiveness to tumors hence supporting tumor growth. In PC patients, tumor progression has also been linked to increased immune suppression [113]. Treg expansion following androgen ablation may be one of the mechanisms responsible for transient immune response after androgen ablation [114]. A trend (*p* = 0.029) between OS and a decrease in Treg suppressive function has been shown in post- *versus* pre-vaccination patients [115]. The prognostic implications of the pretreatment level of Th17 cells compared with regulatory T-cell status in PC patients receiving active whole cell immunotherapy was investigated by Derhovanessian *et al*. [116]. They showed that frequency of CCR4^−^/IL-17^+^/CD4^+^ T-cells pre vaccination inversely correlated with TTP in 23 prostate cancer patients. Responder patients with significant reductions in PSA velocity (PSAV) in response to the immunotherapy (*n* = 9) showed a Th17 profile similar to healthy male controls and significantly different from non-responder patients (*n* = 14) [116]. 

The effects of adding low-dose cyclophosphamide to a cell-based immunotherapy was investigated in mice bearing endogenous prostate tumors (TRAMP model). This study showed that dose and timing of cyclophosphamide with allogeneic GVAX immunotherapy is important for potentiating the efficacy of immunotherapy. Interestingly, T_eff_/T_reg_ ratio increased for CD4^+^ and CD8^+^ suggesting that cyclophosphamide may inhibit Tregs thereby stimulating the effector T cells, functional effect of these data was evidenced by reduced tumor weights when cyclophosphamide was administered before each immunotherapy cycle [65].

### 7.3. Modulation of Tumor Microenvironment to Improve Immune Based Therapies

**Figure 1:**
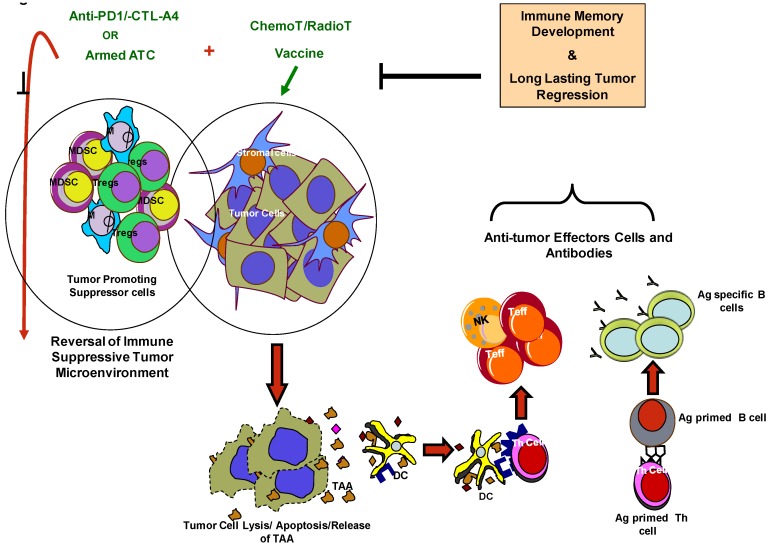
Shows the two key cellular components of the tumor microenvironment (a) tumor component that include tumor cells, stromal cells and cancer stem like cells, and (b) immune component that include cells of the immune system with immune suppressive properties. Therapeutic strategies that can target both components or reverse the immunosuppressive environment and harness the immune cells to target tumor cells would lead to tumor-specific immunological memory for long-lasting regression in cancer patients.

Tumor-derived factors and cellular components such as IL-10 and transforming growth factor-β (TGF-β), IDO, expression of negative co-stimulatory ligands PDL-1 and CTLA-4 and the presence of regulatory lymphocyte and myeloid cell populations pose challenges for the success of immunotherapy and anti-tumor responses [95,117]. Combining vaccines with therapeutic strategies that are designed to inhibit or alleviate the immunosuppressive microenvironment such as imatinib [118] (which inhibits IDO), sunitinib [119] (which antagonizes MDSCs and T_Reg_ cells), cyclophosphamide [120] (kills T_Reg_ cells) and gemcitabine [106] (kills MDSCs) may enhance the effect of immunotherapy and promote anti-tumor immune responses (Figure 1).

## 8. Conclusions

In spite of rapidly progressing treatment modalities for prostate cancer, effective treatment modalities for advanced prostate cancer are still lacking. Although some combination clinical trials in advanced diseases have shown encouraging results, several challenging issues need careful consideration such as timing of immunotherapy, sequence of immunotherapy, androgen deprivation therapy, and rationale for early stage *versus* advanced disease. Perhaps a careful evaluation of existing knowledge may provide a valuable resource to design an effective treatment strategy that can induce tumor-specific immunological memory for long-lasting regression in cancer patients.

## References

[1] Miyamoto H., Messing E.M., Chang C.S. (2004). Androgen deprivation therapy for prostate cancer: Current status and future prospects. Prostate.

[2] Rubin M.A., Maher C.A., Chinnaiyan A.M. (2011). Common gene rearrangements in prostate cancer. J. Clin. Oncol..

[3] Mcneel D.G., Malkovsky M. (2005). Immune-based therapies for prostate cancer. Immunol. Lett..

[4] Fong L., Brockstedt D., Benike C., Breen J.K., Strang G., Ruegg C.L., Engleman E.G. (2001). Dendritic cell-based xenoantigen vaccination for prostate cancer immunotherapy. J. Immunol..

[5] Oesterling J.E. (1991). Prostate specific antigen—A critical-assessment of the most useful tumor-marker for adenocarcinoma of the prostate. J. Urol..

[6] Balk S.P., Ko Y.J., Bubley G.J. (2003). Biology of prostate-specific antigen. J. Clin. Oncol..

[7] Murphy G.P., Greene T.G., Tino W.T., Boynton A.L., Holmes E.H. (1998). Isolation and characterization of monoclonal antibodies specific for the extracellular domain of prostate specific membrane antigen. J. Urol..

[8] Murphy G.P., Elgamal A.A.A., Su S.L., Bostwick D.G., Holmes E.H. (1998). Current evaluation of the tissue localization and diagnostic utility of prostate specific membrane antigen. Cancer.

[9] Solin T., Kontturi M., Pohlmann R., Vihko P. (1990). Gene-Expression and prostate specificity of human prostatic acid-phosphatase (Pap)—Evaluation by rna blot analyses. Biochim. Biophys. Acta.

[10] Fong L., Engleman E.G. (2000). Dendritic cells in cancer immunotherapy. Ann. Rev. Immunol..

[11] Heiser A., Coleman D., Dannull J., Yancey D., Maurice M.A., Lallas C.D., Dahm P., Niedzwiecki D., Gilboa E., Vieweg J. (2002). Autologous dendritic cells transfected with prostate-specific antigen RNA stimulate CTL responses against metastatic prostate tumors. J. Clin. Invest..

[12] Drake C.G. (2010). Prostate cancer as a model for tumour immunotherapy. Nat. Rev. Immunol..

[13] Marrari A., Iero M., Pilla L., Villa S., Salvioni R., Valdagni R., Parmiani G., Rivoltini L. (2007). Vaccination therapy in prostate cancer. Cancer Immunol. Immunother..

[14] Fong L., Benike C., Strang G., Hao Z.M., Smits B., Ruegg C.L., Engleman E.G. (1999). Immunization with dendritic cells pulsed with xenogeneic prostatic acid phosphatase (PAP) induces cellular immune responses in prostate cancer patients. Proc. Am. Assoc. Cancer Res..

[15] Kita H., Matsumura S., He X.S., Ansari A.A., Lian Z.X., de Water J.V., Coppel R.L., Kaplan M.M., Gershwin M.E. (2002). Analysis of substituted peptides of HLA-A2*0201 restricted PDC-E2 peptide CTL epitopes in primary biliary cirrhosis: Implications for immunotherapy. Hepatology.

[16] Pandha H.S., John R.J., Hutchinson J., James N., Whelan M., Corbishley C., Dalgleish A.G. (2004). Dendritic cell immunotherapy for urological cancers using cryopreserved allogeneic tumour lysate-pulsed cells: A phase I/II study. BJU Int..

[17] Small E.J., Fratesi P., Reese D.M., Strang G., Laus R., Peshwa M.V., Valone F.H. (2000). Immunotherapy of hormone-refractory prostate cancer with antigen-loaded dendritic cells. J. Clin. Oncol..

[18] Fong L., Ruegg C.L., Brockstedt D., Engleman E.G., Laus R. (1997). Induction of tissue-specific autoimmune prostatitis with prostatic acid phosphatase immunization—Implications for immunotherapy of prostate cancer. J. Immunol..

[19] Mcneel D.G., Nguyen L.D., Disis M.L. (2001). Identification of T helper epitopes from prostatic acid phosphatase. Cancer Res..

[20] Kaufman H.L., Dipaola R., von Mehren M., Marshall J., Lyerly H.K., Streicher H., Schlom J., Panicali D., Schuetz T. (2004). Safety profile of therapeutic pox virus-based vaccines for cancer. J. Clin. Oncol..

[21] DiPaola R.S., Plante M., Kaufman H., Petrylak D.P., Israeli R., Lattime E., Manson K., Schuetz T. (2006). A phase I trial of pox PSA vaccines (PROSTVAC (R)-VF) with b7-1, ICAM-1, and LFA-3 co-stimulatory molecules (TRICOM (TM)) in patients with prostate cancer. J. Transl. Med..

[22] Arlen P.M., Gulley J.L., Madan R.A., Hodge J.W., Schlom J. (2007). Preclinical and clinical studies of recombinant poxvirus Vaccines for carcinoma therapy. Crit. Rev. Immunol..

[23] Higano C.S., Schellhammer P.F., Small E.J., Burch P.A., Nemunaitis J., Yuh L., Provost N., Frohlich M.W. (2009). Integrated data from 2 randomized, double-blind, placebo-controlled, phase 3 trials of active cellular immunotherapy with Sipuleucel-T in advanced prostate cancer. Cancer.

[24] Madan R.A., Arlen P.M., Mohebtash M., Hodge J.W., Gulley J.L. (2009). Prostvac-VF: A vector-based vaccine targeting PSA in prostate cancer. Expert Opin. Investig. Drugs.

[25] Tjoa B.A., Simmons S.J., Bowes V.A., Ragde H., Rogers M., Elgamal A., Kenny G.M., Cobb O.E., Ireton R.C., Troychak M.J. (1998). Evaluation of phase I/II clinical trials in prostate cancer with dendritic cells and PSMA peptides. Prostate.

[26] Iwasaki A., Medzhitov R. (2004). Toll-like receptor control of the adaptive immune responses. Nat. Immunol..

[27] Iwasaki A., Medzhitov R. (2010). Regulation of adaptive immunity by the innate immune system. Science.

[28] Graddis T.J., McMahan C.J., Tamman J., Page K.J., Trager J.B. (2011). Prostatic acid phosphatase expression in human tissues. Int. J. Clin. Exp. Pathol..

[29] Nelson P.S. (2002). Identifying immunotherapeutic targets for prostate carcinoma through the analysis of gene expression profiles. Ann. NY Acad. Sci..

[30] Small E.J., Schellhammer P.F., Higano C.S., Redfern C.H., Nemunaitis J.J., Valone F.H., Verjee S.S., Jones L.A., Hershberg R.M. (2006). Placebo-controlled phase III trial of immunologic therapy with sipuleucel-T (APC8015) in patients with metastatic, asymptomatic hormone refractory prostate cancer. J. Clin. Oncol..

[31] Kantoff P.W., Higano C.S., Shore N.D., Berger E.R., Small E.J., Penson D.F., Redfern C.H., Ferrari A.C., Dreicer R., Sims R.B. (2010). Sipuleucel-T immunotherapy for castration-resistant prostate cancer. N. Engl. J. Med..

[32] Sheikh N.A., Petrylak D., Kantoff P.W., dela Rosa C., Stewart F.P., Kuan L.Y., Whitmore J.B., Trager J.B., Poehlein C.H., Frohlich M.W. (2013). Sipuleucel-T immune parameters correlate with survival: An analysis of the randomized phase 3 clinical trials in men with castration-resistant prostate cancer. Cancer Immunol. Immunother..

[33] Dranoff G., Jaffee E., Lazenby A., Golumbek P., Levitsky H., Brose K., Jackson V., Hamada H., Pardoll D., Mulligan R.C. (1993). Vaccination with irradiated tumor-cells engineered to secrete murine granulocyte-macrophage colony-stimulating factor stimulates potent, specific, and long-lasting antitumor immunity. Proc. Natl. Acad. Sci. USA.

[34] Ward J.E., Mcneel D.G. (2007). GVAX: An allogeneic, whole-cell, GM-CSF-secreting cellular immunotherapy for the treatment of prostate cancer. Expert Opin. Biol. Ther..

[35] Simons J.W., Carducci M.A., Mikhak B., Lim M., Biedrzycki B., Borellini F., Clift S.M., Hege K.M., Ando D.G., Piantadosi S. (2006). Phase I/II trial of an allogeneic cellular immunotherapy in hormone-naive prostate cancer. Clin. Cancer Res..

[36] Small E.J., Sacks N., Nemunaitis J., Urba W.J., Dula E., Centeno A.S., Nelson W.G., Ando D., Howard C., Borellini F. (2007). Granulocyte macrophage colony-stimulating factor-secreting allogeneic cellular immunotherapy for hormone-refractory prostate cancer. Clin. Cancer Res..

[37] Higano C.S., Corman J.M., Smith D.C., Centeno A.S., Steidle C.P., Gittleman M., Simons J.W., Sacks N., Aimi J., Small E.J. (2008). Phase 1/2 dose-escalation study of a GM-CSF-Secreting, allogeneic, cellular immunotherapy for metastatic hormone-refractory prostate cancer. Cancer.

[38] Higano C., Saad F., Somer B., Curti B., Petrylak D., Drake C.G., Schnell F., Redfern C.H., Schrijvers D., Sacks N. A phase III trial of GVAX immunotherapy for prostate cancer *versus* docetaxel plus prednisone in asymptomatic, castration-resistant prostate cancer (CRPC). ASCO 2009 Genitourinary Cancer Symposium.

[39] Van den Eertwegh A.J.M., Versluis J., van den Berg H.P., Santegoets S.J.A.M., van Moorselaar R.J.A., van der Sluis T.M., Gall H.E., Harding T.C., Jooss K., Lowy I. (2012). Combined immunotherapy with granulocyte-macrophage colony-stimulating factor-transduced allogeneic prostate cancer cells and ipilimumab in patients with metastatic castration-resistant prostate cancer: A phase 1 dose-escalation trial. Lancet Oncol..

[40] Eder J.P., Kantoff P.W., Roper K., Xu G.X., Bubley G.J., Boyden J., Gritz L., Mazzara G., Oh W.K., Arlen P. (2000). A phase I trial of a recombinant vaccinia virus expressing prostate-specific antigen in advanced prostate cancer. Clin. Cancer Res..

[41] Sanda M.G., Smith D.C., Charles L.G., Hwang C., Pienta K.J., Schlom J., Milenic D., Panicali D., Montie J.E. (1999). Recombinant vaccinia-PSA [PROSTVAC] can induce a prostate-specific immune response in androgen-modulated human prostate cancer. Urology.

[42] Hodge J.W., McLaughlin J.P., Kantor J.A., Schlom J. (1997). Diversified prime and boost protocols using recombinant vaccinia virus and recombinant non-replicating avian pox virus to enhance T-cell immunity and antitumor responses. Vaccine.

[43] Gulley J., Chen A.P., Dahut W., Arlen P.M., Bastian A., Steinberg S.M., Tsang K., Panicali D., Poole D., Schlom J. (2002). Phase I study of a vaccine using recombinant vaccinia virus expressing PSA (rV-PSA) in patients with metastatic androgen-independent prostate cancer. Prostate.

[44] Kaufman H.L., Wang W., Manola J., DiPaola R.S., Ko Y.J., Sweeney C., Whiteside T.L., Schlom J., Wilding G., Weiner L.M. (2004). Phase II randomized study of vaccine treatment of advanced prostate cancer (E7897): A trial of the Eastern Cooperative Oncology group. J. Clin. Oncol..

[45] Kantoff P.W., Schuetz T.J., Blumenstein B.A., Glode L.M., Bilhartz D.L., Wyand M., Manson K., Panicali D.L., Laus R., Schlom J. (2010). Overall survival analysis of a phase II randomized controlled trial of a poxviral-based PSA-Targeted immunotherapy in metastatic castration-resistant prostate cancer. J. Clin. Oncol..

[46] Madan R.A., Mohebtash M., Arlen P.M., Vergati M., Rauckhorst M., Steinberg S.M., Tsang K.Y., Poole D.J., Parnes H.L., Wright J.J. (2012). Ipilimumab and a poxviral vaccine targeting prostate-specific antigen in metastatic castration-resistant prostate cancer: A phase 1 dose-escalation trial. Lancet Oncol..

[47] Antonarakis E.S. (2012). Combining active immunotherapy with immune checkpoint blockade for the treatment of advanced prostate cancer. Asian J. Androl..

[48] Johnson L.E., Frye T.P., Chinnasamy N., Chinnasamy D., Mcneel D.G. (2007). Plasmid DNA vaccine encoding prostatic acid phosphatase is effective in eliciting autologous antigen-specific CD8+ T cells. Cancer Immunol. Immunother..

[49] Hawkins W.G., Gold J.S., Blachere N.E., Bowne W.B., Hoos A., Lewis J.J., Houghton A.N. (2002). Xenogeneic DNA immunization in melanoma models for minimal residual disease. J. Surg. Res..

[50] Johnson L.E., Frye T.P., Arnot A.R., Marquette C., Couture L.A., Gendron-Fitzpatrick A., Mcneel D.G. (2006). Safety and immunological efficacy of a prostate cancer plasmid DNA vaccine encoding prostatic acid phosphatase (PAP). Vaccine.

[51] Pavlenko M., Roos A.K., Lundqvist A., Palmborg A., Miller A.M., Ozenci V., Bergman B., Egevad L., Hellstrom M., Kiessling R. (2004). A phase I trial of DNA vaccination with a plasmid expressing prostate-specific antigen in patients with hormone-refractory prostate cancer. Br. J. Cancer.

[52] Mcneel D.G., Dunphy E.J., Davies J.G., Frye T.P., Johnson L.E., Staab M.J., Horvath D.L., Straus J., Alberti D., Marnocha R. (2009). Safety and immunological efficacy of a DNA vaccine encoding prostatic acid phosphatase in patients with stage D0 prostate cancer. J. Clin. Oncol..

[53] Becker J.T., Olson B.M., Johnson L.E., Davies J.G., Dunphy E.J., Mcneel D.G. (2010). DNA Vaccine Encoding Prostatic Acid Phosphatase (PAP) elicits long-term t-cell responses in patients with recurrent prostate cancer. J. Immunother..

[54] Sanchez K.M., Sweeney C.J., Mass R., Koch M.O., Eckert G.J., Geary W.A., Baldridge L.A., Zhang S.B., Eble J.N., Cheng L. (2002). Evaluation of HER-2/neu expression in prostatic adenocarcinoma: A request for a standardized, organ specific methodology. Cancer.

[55] Visakorpi T., Kallioniemi O.P., Koivula T., Harvey J., Isola J. (1992). Expression of epidermal growth-factor receptor and Erbb2 (Her-2/Neu) oncoprotein in prostatic carcinomas. Modern Pathol..

[56] Bartlett J.M.S., Brawley D., Grigor K., Munro A.F., Dunne B., Edwards J. (2005). Type I receptor tyrosine kinases are associated with hormone escape in prostate cancer. J. Pathol..

[57] Nishio Y., Yamada Y., Kokubo H., Nakamura K., Aoki S., Taki T., Honda N., Nakagawa A., Saga S., Hara K. (2006). Prognostic significance of immunohistochemical expression of the HER-2/neu oncoprotein in bone metastatic prostate cancer. Urology.

[58] Davol P.A., Smith J.A., Kouttab N., Elfenbein G.J., Lum L.G. (2004). Anti-CD3 × Anti-HER2 bispecific antibody effectively redirects armed T cells to inhibit tumor development and growth in hormone-refractory prostate cancer-bearing SCID-Beige mice. Clin. Prostate Cancer.

[59] Grabert R.C., Cousens L.P., Smith J.A., Olson S., Gall J., Young W.B., Davol P.A., Lum L.G. (2006). Human T cells armed with Her2/neu bispecific antibodies divide, are cytotoxic, and secrete cytokines with repeated stimulation. Clin. Cancer Res..

[60] Lum H.E., Miller M., Davol P.A., Grabert R.C., Davis J.B., Lum L.G. (2005). Preclinical studies comparing different bispecific antibodies for redirecting T cell cytotoxicity to extracellular antigens on prostate carcinomas. Anticancer Res..

[61] Ricciardelli C., Jackson M.W., Choong C.S., Stahl J., Marshall V.R., Horsfall D.J., Tilley W.D. (2008). Elevated levels of HER-2/neu and androgen receptor in clinically localized prostate cancer identifies metastatic potential. Prostate.

[62] Lum L.G. (2013).

[63] Wada S., Yoshimura K., Hipkiss E.L., Harris T.J., Yen H.R., Goldberg M.V., Grosso J.F., Getnet D., DeMarzo A.M., Netto G.J. (2009). Cyclophosphamide augments antitumor immunity: Studies in an autochthonous prostate cancer model. Cancer Res..

[64] Ghiringhelli F., Apetoh L., Tesniere A., Aymeric L., Ma Y.T., Ortiz C., Vermaelen K., Panaretakis T., Mignot G., Ullrich E. (2009). Activation of the NLRP3 inflammasome in dendritic cells induces IL-1 beta-dependent adaptive immunity against tumors. Nat. Med..

[65] Green D.R., Ferguson T., Zitvogel L., Kroemer G. (2009). Immunogenic and tolerogenic cell death. Nat. Rev. Immunol..

[66] Apetoh L., Ghiringhelli F., Tesniere A., Obeid M., Ortiz C., Criollo A., Mignot G., Maiuri M.C., Ullrich E., Saulnier P. (2007). Toll-like receptor 4-dependent contribution of the immune system to anticancer chemotherapy and radiotherapy. Nat. Med..

[67] Arlen P.M., Gulley J.L., Parker C., Skarupa L., Pazdur M., Tsang K.Y., Schlom J., Dahut W.L. (2006). A randomized pilot study of concurrent docetaxel plus vaccine *versus* vaccine alone in metastatic androgen independent prostate cancer. J. Clin. Oncol..

[68] Gulley J.L., Drake C.G. (2011). Immunotherapy for prostate cancer: Recent advances, lessons learned, and areas for further research. Clin. Cancer Res..

[69] Nesslinger N.J., Ng A., Tsang K.Y., Ferrara T., Schlom J., Gulley J.L., Nelson B.H. (2010). A viral vaccine encoding prostate-specific antigen induces antigen spreading to a common set of self-proteins in prostate cancer patients. Clin. Cancer Res..

[70] Small E., Demkow T., Gerritsen W.R., Rolland F., Hoskin P., Smith D.C., Parker C., Chondros D., Ma J., Hege K. A Phase III trial of GVAX Immunotherapy for Prostate Cancer in Combination with Docetaxel *versus* Docetaxel Plus Prednisone in Symptomatic, Castration-Resistant Prostate Cancer (CRPC). ASCO 2009 Genitourinary Cancer Symposium.

[71] Antonarakis E.S., Drake C.G. (2010). Current status of immunological therapies for prostate cancer. Curr. Opin. Urol..

[72] Sharpe A.H., Abbas A.K. (2006). Focus on research: T-cell costimulation—Biology, therapeutic potential, and challenges. N. Engl. J. Med..

[73] Parry R.V., Chemnitz J.M., Frauwirth K.A., Lanfranco A.R., Braunstein I., Kobayashi S.V., Linsley P.S., Thompson C.B., Riley J.L. (2005). CTLA-4 and PD-1 receptors inhibit T-cell activation by distinct mechanisms. Mol. Cell Biol..

[74] O’Day S.J., Hamid O., Urba W.J. (2007). Targeting cytotoxic T-lymphocyte antigen-4 (CTLA-4)—A novel strategy for the treatment of melanoma and other malignancies. Cancer.

[75] Krummel M.F., Allison J.P. (1995). Cd28 and Ctla-4 have opposing effects on the response of T-Cells to stimulation. J. Exp. Med..

[76] Miller A.M., Pisa P. (2007). Tumor escape mechanisms in prostate cancer. Cancer Immunol. Immunother..

[77] Hurwitz A.A., Yu T.F.Y., Leach D.R., Allison J.P. (1998). CTLA-4 blockade synergizes with tumor-derived granulocyte-macrophage colony-stimulating factor for treatment of an experimental mammary carcinoma. Proc. Natl. Acad. Sci. USA.

[78] Hurwitz A.A., Foster B.A., Kwon E.D., Greenberg N.M., Allison J.P. (1999). Immunotherapy for prostate cancer in the TRAMP model using a GM-CSF-expressing vaccine and CTLA-4 blockade. FASEB J..

[79] Small E.J., Tchekmedyian N.S., Rini B.I., Fong L., Lowy I., Allison J.P. (2007). A pilot trial of CTLA-4 blockade with human anti-CTLA-4 in patients with hormone-refractory prostate cancer. Clin. Cancer Res..

[80] Fong L., Kwek S.S., O’Brien S., Kavanagh B., Mcneel D.G., Weinberg V., Lin A.M., Rosenberg J., Ryan C.J., Rini B.I. (2009). Potentiating endogenous antitumor immunity to prostate cancer through combination immunotherapy with CTLA4 blockade and GM-CSF. Cancer Res..

[81] Kwek S.S., Dao V., Roy R., Hou Y.F., Alajajian D., Simko J.P., Small E.J., Fong L. (2012). Diversity of antigen-specific responses induced *in vivo* with CTLA-4 blockade in prostate cancer patients. J. Immunol..

[82] Madan R.A., Mohebtash M., Arlen P.M., Vergati M., Steinberg S.M., Tsang K.Y., Dahut W.L., Schlom J., Gulley J.L. (2010). Overall survival (OS) analysis of a phase l trial of a vector-based vaccine (PSA-TRICOM) and ipilimumab (Ipi) in the treatment of metastatic castration-resistant prostate cancer (mCRPC). J. Clin. Oncol..

[83] Gerritsen W.R., van den Eertwegh A.J.M., de Gruijl T.D., Giaccone G., Scheper R.J., Sacks N., Harding T., Lowy I., Stankevich E., Hege K. (2007). Biochemical and immunologic correlates of clinical response in a combination trial of the GM-CSF-gene transduced allogeneic prostate cancer immunotherapy and ipilimumab in patients with metastatic hormone-refractory prostate cancer (mHRPC). ASCO Meeting Abstr..

[84] Freeman G.J., Long A.J., Iwai Y., Bourque K., Chernova T., Nishimura H., Fitz L.J., Malenkovich N., Okazaki T., Byrne M.C. (2000). Engagement of the PD-1 immunoinhibitory receptor by a novel B7 family member leads to negative regulation of lymphocyte activation. J. Exp. Med..

[85] Hirano F., Kaneko K., Tamura H., Dong H.D., Wang S.D., Ichikawa M., Rietz C., Flies D.B., Lau J.S., Zhu G.F. (2005). Blockade of B7-H1 and PD-1 by monoclonal antibodies potentiates cancer therapeutic immunity. Cancer Res..

[86] Freeman G.J., Long A.J., Iwai Y., Bourque K., Chernova T., Nishimura H., Fitz L., Malenkovich N., Okazaki T., Byrne M. (2000). The B7-homologue, PD-L, is the ligand of the PD-1 immunoinhibitory receptor. FASEB J..

[87] Hamanishi J., Mandai M., Iwasaki M., Okazaki T., Tanaka Y., Yamaguchi K., Higuchi T., Yagi H., Takakura K., Minato N. (2007). Programmed cell death 1 ligand 1 and tumor-infiltrating CD8(+) T lymphocytes are prognostic factors of human ovarian cancer. Proc. Natl. Acad. Sci. USA.

[88] Sfanos K.S., Bruno T.C., Meeker A.K., de Marzo A.M., Isaacs W.B., Drake C.G. (2009). Human prostate-infiltrating CD8(+) T lymphocytes are oligoclonal and PD-I+. Prostate.

[89] Brahmer J.R., Drake C.G., Wollner I., Powderly J.D., Picus J., Sharfman W.H., Stankevich E., Pons A., Salay T.M., McMiller T.L. (2010). Phase I Study of Single-Agent Anti-Programmed Death-1 (MDX-1106) in Refractory Solid Tumors: Safety, Clinical Activity, Pharmacodynamics, and Immunologic Correlates. J. Clin. Oncol..

[90] Topalian S.L., Hodi F.S., Brahmer J.R., Gettinger S.N., Smith D.C., McDermott D.F., Powderly J.D., Carvajal R.D., Sosman J.A., Atkins M.B. (2012). Safety, Activity, and Immune Correlates of Anti-PD-1 Antibody in Cancer. N. Engl. J. Med..

[91] Brahmer J.R., Tykodi S.S., Chow L.Q.M., Hwu W.J., Topalian S.L., Hwu P., Drake C.G., Camacho L.H., Kauh J., Odunsi K. (2012). Safety and activity of Anti-PD-L1 antibody in patients with advanced cancer. N. Engl. J. Med..

[92] Drake C.G., Jaffee E., Pardoll D.M. (2006). Mechanisms of immune evasion by tumors. Adv. Immunol..

[93] Tien A.H., Xu L.X., Helgason C.D. (2005). Altered immunity accompanies disease progression in a mouse model of prostate dysplasia. Cancer Res..

[94] Miller A.M., Lundberg K., Ozenci V., Banham A.H., Hellstrom M., Egevad L., Pisa P. (2006). CD4(+)CD25(high) T cells are enriched in the tumor and peripheral blood of prostate cancer patients. J. Immunol..

[95] Rabinovich G.A., Gabrilovich D., Sotomayor E.M. (2007). Immunosuppressive strategies that are mediated by tumor cells. Ann. Rev. Immunol..

[96] Muller A.J., Prendergast G.C. (2005). Marrying immunotherapy with chemotherapy: Why say IDO?. Cancer Res..

[97] Yu H., Pardoll D., Jove R. (2009). STATs in cancer inflammation and immunity: A leading role for STAT3. Nat. Rev. Cancer.

[98] Malachowski W.P., Metz R., Prendergast G.C., Muller A.J. (2005). A new cancer immunosuppression target: Indoleamine 2,3-dioxygenase (IDO). A review of the IDO mechanism, inhibition and therapeutic applications. Drugs Future.

[99] Gabrilovich D.I. (2009). Myeloid-derived suppressor cells and tumor microenvironment. J. Immunother..

[100] Gabrilovich D.I., Nagaraj S. (2009). Myeloid-derived suppressor cells as regulators of the immune system. Nat. Rev. Immunol..

[101] Bronte V. (2007). Myeloid-derived suppressor cells in cancer. J. Immunother..

[102] Kozin S.V., Kamoun W.S., Huang Y.H., Dawson M.R., Jain R.K., Duda D.G. (2010). Recruitment of myeloid but not endothelial precursor cells facilitates tumor regrowth after local irradiation. Cancer Res..

[103] Levy D.E., Darnell J.E. (2002). STATs: Transcriptional control and biological impact. Nat. Rev. Mol. Cell Biol..

[104] Vuk-Pavlovic S., Bulur P.A., Lin Y., Qin R., Szumlanski C.L., Zhao X.H., Dietz A.B. (2010). Immunosuppressive CD14(+)HLA-DRlow/-monocytes in prostate cancer. Prostate.

[105] Gustafson M.P., Lin Y., League S.C., Bulur P.A., Abraham R.S., Vuk-Pavlovic S., Gastineau D.A., Dietz A.B. (2010). Loss of HLA-DR Expression on CD14+Cells; a common marker of immunosuppression in cancer patients. J. Immunother..

[106] Suzuki E., Kapoor V., Jassar A.S., Kaiser L.R., Albelda S.M. (2005). Gemcitabine selectively eliminates splenic Gr-1(+)/CD11b(+) myeloid suppressor cells in tumor-bearing animals and enhances antitumor immune activity. Clin. Cancer Res..

[107] Le H.K., Graham L., Cha E., Morales J.K., Manjili M.H., Bear H.D. (2009). Gemcitabine directly inhibits myeloid derived suppressor cells in BALB/c mice bearing 4T1 mammary carcinoma and augments expansion of T cells from tumor-bearing mice. Int. Immunopharmacol..

[108] Pili R., Haggman M., Stadler W.M., Gingrich J.R., Assikis V.J., Bjork A., Nordle O., Forsberg G., Carducci M.A., Armstrong A.J. (2011). Phase II randomized, double-blind, placebo-controlled study of tasquinimod in men with minimally symptomatic metastatic castrate-resistant prostate cancer. J. Clin. Oncol..

[109] Bjork P., Bjork A., Vogl T., Stenstrom M., Liberg D., Olsson A., Roth J., Ivars F., Leanderson T. (2009). Identification of human S100A9 as a novel target for treatment of autoimmune disease via binding to Quinoline-3-Carboxamides. PLoS Biol..

[110] Murdoch C., Muthana M., Coffelt S.B., Lewis C.E. (2008). The role of myeloid cells in the promotion of tumour angiogenesis. Nat. Rev. Cancer.

[111] Thakur A., Schalk D., Tomaszewski E., Kondadasula S.V., Yano H., Sarkar F.H., Lum L.G. (2013). Microenvironment generated during EGFR targeted killing of pancreatic tumor cells by ATC inhibits myeloid-derived suppressor cells through COX2 and PGE2 dependent pathway. J. Transl. Med..

[112] Thakur A., Schalk D., Sarkar S.H., Al-Khadimi Z., Sarkar F.H., Lum L.G. (2012). A Th1 cytokine-enriched microenvironment enhances tumor killing by activated T cells armed with bispecific antibodies and inhibits the development of myeloid-derived suppressor cells. Cancer Immunol. Immunother..

[113] Hiura T., Kagamu H., Miura S., Ishida A., Tanaka H., Tanaka J., Gejyo F., Yoshizawa H. (2005). Both regulatory T cells and antitumor effector T cells are primed in the same draining lymph nodes during tumor progression. J. Immunol..

[114] Tang S., Moore M.L., Grayson J.M., Dubey P. (2012). Increased CD8(+) T-cell function following castration and immunization is countered by parallel expansion of regulatory T cells. Cancer Res..

[115] Vergati M., Cereda V., Madan R.A., Gulley J.L., Huen N.Y., Rogers C.J., Hance K.W., Arlen P.M., Schlom J., Tsang K.Y. (2011). Analysis of circulating regulatory T cells in patients with metastatic prostate cancer pre- *versus* post-vaccination. Cancer Immunol. Immunother..

[116] Derhovanessian E., Adams V., Hahnel K., Groeger A., Pandha H., Ward S., Pawelec G. (2009). Pretreatment frequency of circulating IL-17(+)CD4(+) T-cells, but not Tregs, correlates with clinical response to whole-cell vaccination in prostate cancer patients. Int. J. Cancer.

[117] Vanneman M., Dranoff G. (2012). Combining immunotherapy and targeted therapies in cancer treatment. Nat. Rev. Cancer.

[118] Balachandran V.P., Cavnar M.J., Zeng S., Bamboat Z.M., Ocuin L.M., Obaid H., Sorenson E.C., Popow R., Ariyan C., Rossi F. (2011). Imatinib potentiates antitumor T cell responses in gastrointestinal stromal tumor through the inhibition of Ido. Nat. Med..

[119] Ozao-Choy J., Ma G., Kao J., Wang G.X., Meseck M., Sung M., Schwartz M., Divino C.M., Pan P.Y., Chen S.H. (2009). The novel role of tyrosine kinase inhibitor in the reversal of immune suppression and modulation of tumor microenvironment for immune-based cancer therapies. Cancer Res..

[120] Ghiringhelli F., Larmonier N., Schmitt E., Parcellier A., Cathelin D., Garrido C., Chauffert B., Solary E., Bonnotte B., Martin F. (2004). CD4(+)CD25(+) regulatory T cells suppress tumor immunity but are sensitive to cyclophosphamide which allows immunotherapy of established tumors to be curative. Eur. J. Immunol..

